# Second-line monotherapy with a PD-1/CTLA-4 inhibitor effectively treated multiple brain and lung metastases of cervical cancer: a case report

**DOI:** 10.3389/fimmu.2024.1434697

**Published:** 2024-10-14

**Authors:** Juan Ni, Xiaoyue Dong, Huafeng Shou, Qing Xu, Zhuomin Yin, Hanmei Lou

**Affiliations:** ^1^ Department of Gynecologic Oncology, Zhejiang Cancer Hospital, Hangzhou, China; ^2^ Department of Gynecology, Ruian Maternity and Child Care Hospital, Wengzhou, China; ^3^ Department of Gynecology, Zhejiang Provincial People’s Hospital, Hangzhou, China; ^4^ Zhejiang Chinese Medical University, Hangzhou, China

**Keywords:** brain metastasis, cervical cancer, PD-1/CTLA-4 inhibitor, immunotherapy, case report

## Abstract

**Background:**

Brain metastasis (BM) from cervical cancer (CC) is extremely rare. The prognosis of BM is poor. To our knowledge, no satisfactory therapeutic and standard effective treatments have been established. Immune checkpoint inhibitors (ICIs) treatment is emerging as a promising treatment in recurrence and metastasis(B/M) cervical cancer in recent years.

**Case:**

We present a 50-year-old patient with CC who developed multiple metastasis (lung, brain and skin) 2 years after postoperative chemoradiotherapy. She received palliative therapy included chemotherapy, resection and stereotactic radiosurgery of BM with poor response. Then, the patient received second-line palliative monotherapy with a PD-1/CTLA-4 inhibitor(cadonilimab) and achieved clinical very good partial response (VGPR), a progression-free survival (PFS) of 14 months and overall survival of more than 18 months since BM.

**Conclusion:**

We report a case of cervical cancer with multiple metastasis receiving cadonilimab and achieved considerable response and survival benefit.

## Introduction

Cervical cancer (CC) ranks fifth in incidence among malignancies in female patients in China (1). According to the 2022 Disease Survey, while its incidence decreased gradually, its mortality rate is increasing (1). It is rare for these tumors to metastasize to the brain and skin. Patients with brain metastasis (BM) have a greater risk of death than those without BM (2).

However, treatment options remain limited. In recent years, immune checkpoint inhibitors (ICIs) have shown substantial clinical benefits in the treatment of CC. Cadonilimab is a first-in-class bispecific antibody that targets both PD-1 and CTLA-4. In CC studies (3, 4), cadonilimab showed encouraging activity in the treatment of recurrent and metastatic (R/M) CC. Nevertheless, ICIs outcomes are unsatisfactory for CC patients with BM.

We present the clinical experience of a woman with CC with BM who had a notable response to treatment with cadonilimab.

## Case report

**Figure 1:**
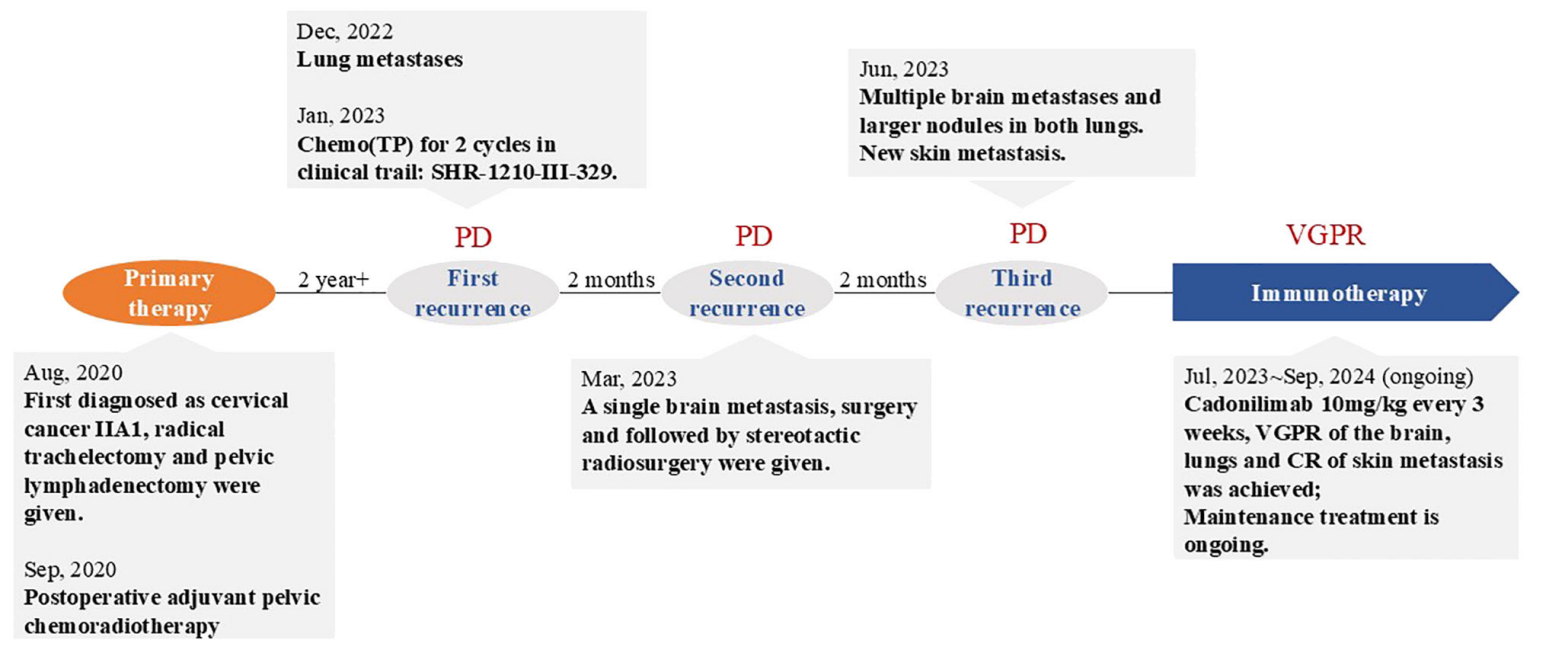
Timeline of diagnose and treatment of the patient.

**Figure 2:**
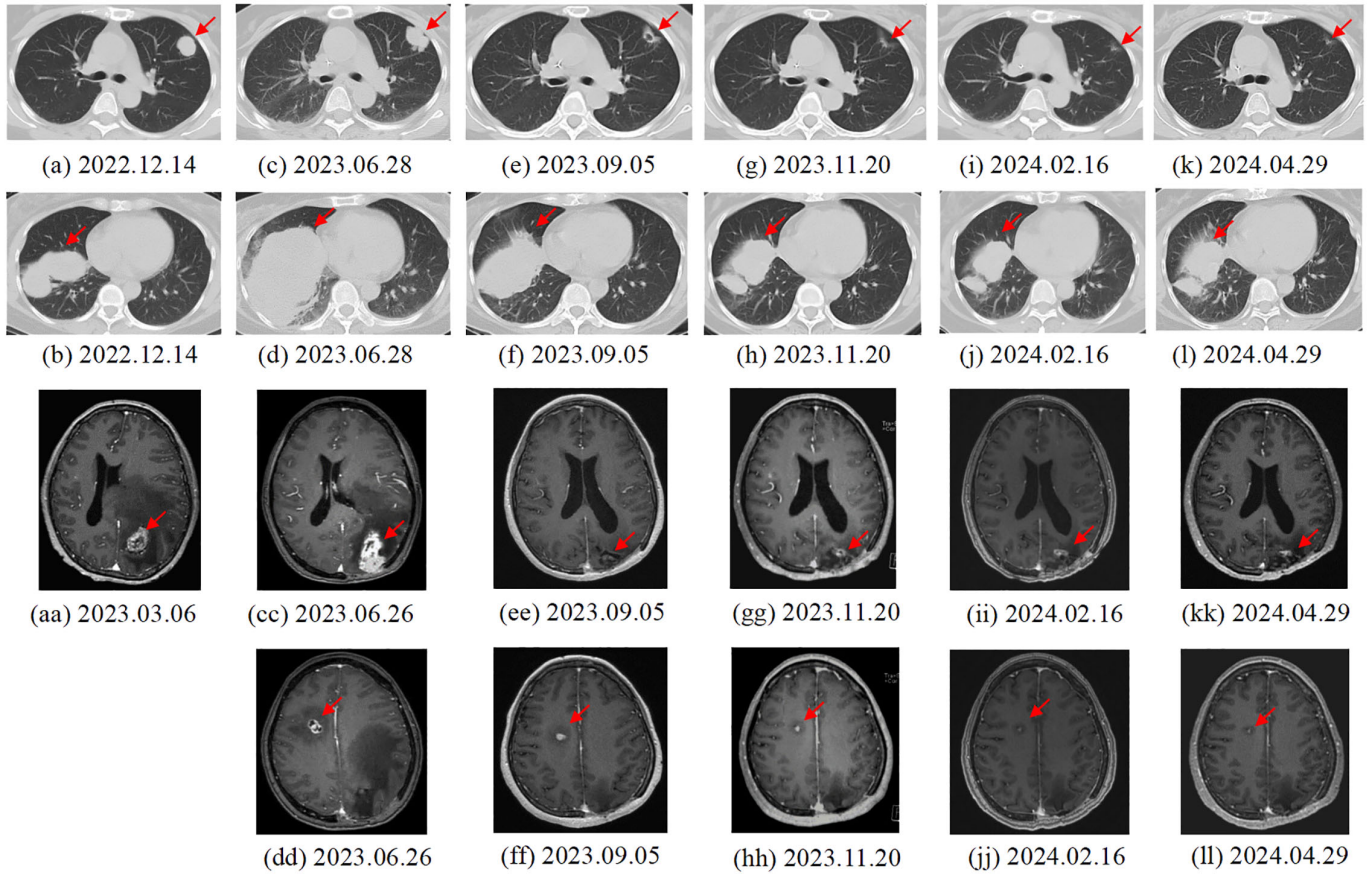
Changes in lung **(a-l)** and brain **(aa-ll)** metastases of the patient. **(A, B)** 2022.12.14. First recurrence: Results of chest CT scan. The largest tumor was 6.0 × 4.0 cm in size. **(C, D)** 2023.6.28. Third recurrence: Results of chest CT scan. The largest tumor was 10.6 × 6.9 cm in size. **(E, F)** 2023.9.05. After two cycles of cadonilimab: Results of chest CT scan. The largest tumor was 8.0 × 5.0 cm in size. **(G, H)** 2023.11.20. After five cycles of cadonilimab: Results of chest CT scan. **(I, J)** 2024.02.16. maintenance treatment of cadonilimab: Results of chest CT scan. The largest tumor was 5.8 × 2.7 cm in size. **(K, L)** 2024.04.29. maintenance treatment of cadonilimab: Results of chest CT scan. The largest tumor was 5.2 × 2.6 cm in size. **(aa)** 2023.3.06. Second recurrence: MRI and CT of the brain showed a single 3.6 × 2.5 cm metastasis within the left supratentorial lobe. **(CC, DD)** 2023.6.26. Third recurrence: Multiple brain metastases in the parenchyma on both sides. **(EE, FF)** 2023.9.05. After two cycles of cadonilimab: Results of brain MRI scan. The interval shank with largest tumor was 1.5× 0.8 cm in size (2e). **(GG, HH)** 2023.11.20. After five cycles of cadonilimab: Results of brain MRI scan. The interval shank with largest tumor was 1.1×0. 8 cm in size (2f). **(II, JJ)** 2024.02.16. maintenance treatment of cadonilimab: Results of brain MRI scan. The interval shank with largest tumor was 0.9×0. 7 cm in size. **(KK, LL)** 2024.04.29. maintenance treatment of cadonilimab: Results of brain MRI scan. The interval shank with largest tumor was 0.9×0. 7 cm in size.

A 50-year-old adult patient with CC (initially diagnosed as FIGO2018 stage IIA1) reported cough 2 years after postoperative chemoradiotherapy in Dec, 2022 (Timeline illustrated in **Figure 1**). Computed tomography (CT) of the thorax revealed multiple spaces occupying both lungs (**Figure 2A**). The largest one was in the right lower lobe and measured 4×6 cm (**Figure 2B**). A needle biopsy revealed squamous cell carcinoma with P16+ which supported the diagnosis of metastasis from CC. No genetic, family, or psychosocial history were reported. The patient then took part in SHR-1210-III-329 clinical trial (ClinicalTrials.gov identifier NCT04906993) in Jan 2023. The patient was randomly assigned to the chemo group and received chemotherapy (paclitaxel 175 mg/m^2^ and cisplatin 50 mg/m^2^) every three weeks. Shortly after two cycles, the patient developed acute right hemiballismus and a cerebral hernia in Feb 2023. The Eastern Cooperative Oncology Group (ECOG) performance status score was 3. Magnetic resonance imaging (MRI) of the brain revealed a single, well-circumscribed peripheral-enhancing lesion measuring 3.6×2.5 cm within the left supratentorial lobe with surrounding vasogenic edema (**Figure 2AA**).

**Figure 3:**
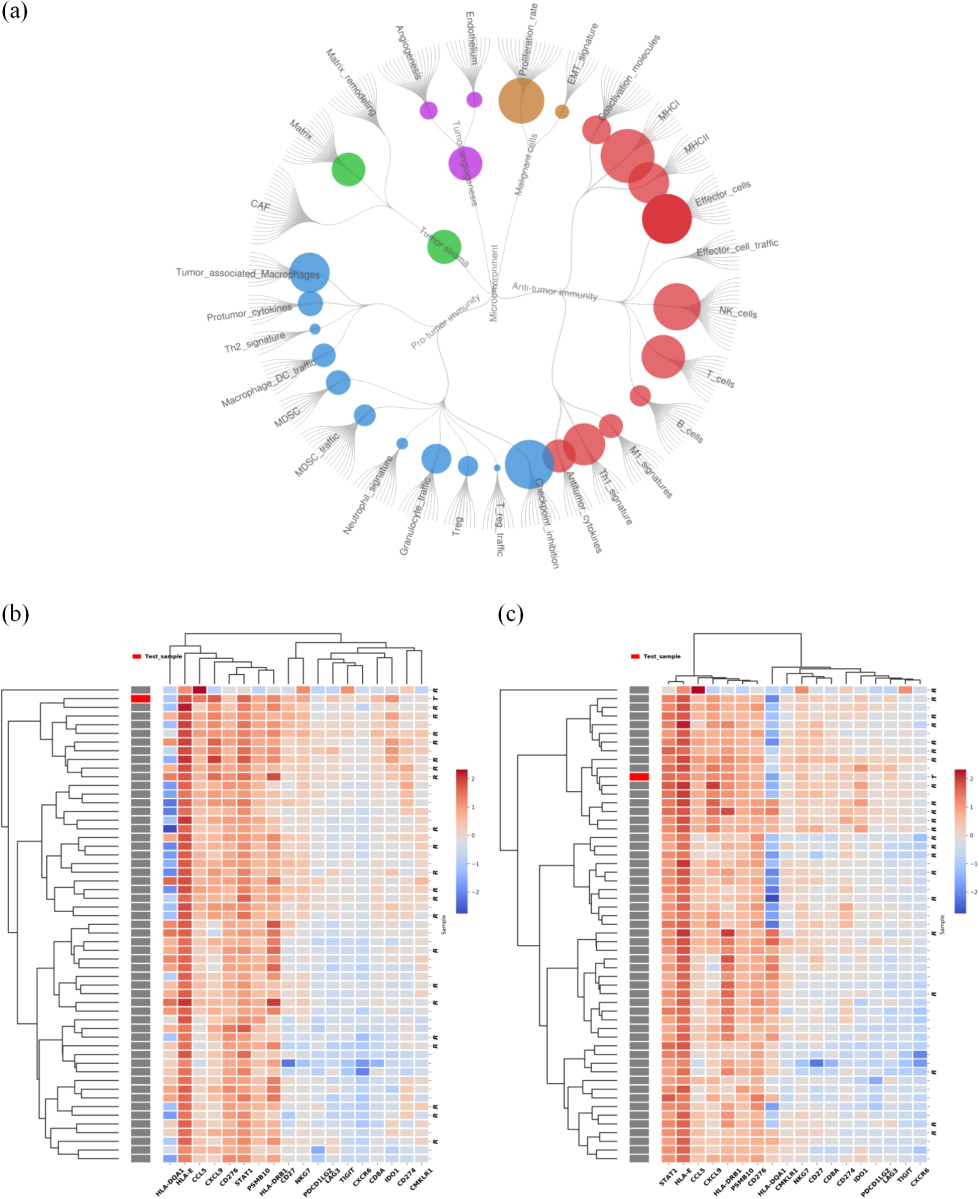
Results of tumor immune microenvironment (TIME) analysis based on a 2660-gene RNA-sequencing. **(A)** Immunoenriched/nonfibrotic subtype (IE) type of CC and BM lesion. **(B)** The GEP score of the cervical tumor was 12.2. **(C)** The GEP score of BM was 9.2. We employed planetary schema termed Molecular-Functional portrait (MF Portrait) to define the hot and cold tumor as follow: First, we calculated ssGSEA score of 29 immune-associated hallmarks and then normalized by median scaling across all samples. Next Louvain clustering analysis was performed to create the four subtypes, including Immune-Enriched/Fibrotic (IE/F), Immune-Enriched/Non-Fibrotiv(IE), Fibrotic(F), Depleted(D) subtypes. According the Immune enriched characteristics, the IE/F and IE subtypes were combined and labeled as ‘hot’, while the F and D subtypes were collectively termed ‘cold’. The scripts utilized are available at https://github.com/BostonGene (6).

The patient underwent gross total resection of the left supratentorial mass in Mar, 2023, followed by stereotactic radiosurgery (SRS) (21 Gy/3F). Histologically, the metastatic brain tumor was confirmed to be metastasized from the cervix. Immunohistochemistry revealed a combined positive score (CPS) ≥ 1 for programmed death ligand-1 (PD-L1) in both the lung and brain. Using the Master panel (Amoydx, Xiamen), mutations in 571 tumor-related genes were detected at the DNA level and the expression of 2660 genes was analyzed at the RNA level. Raw reads were aligned to hg19 and gene assignment was based on GRCh37.75. The normalized log2(TPM+1) matrix was evaluated using a prior prediction model for RNA-based tumor immune microenvironment (TIME) analysis. The results showed that CC and BM samples revealed a high clonal tumor mutation burden (TMB) (38.33 and 69.766 mutations per megabase) and MSI-H status (MSI score of 10.34 and 31.9). The TIME subtype analysis suggested the presence of ‘hot’ label as immune-enriched, nonfibrotic phenotype (**Figure 3A**) (5). The tumor immune gene expression profile (GEP) scores of CC and BM were 12.2 and 9.2 (**Figures 3B, C**) (5). Rehabilitation treatment after surgery was received. Two months later, the patient suddenly fell into a light coma and could not walk anymore. The ECOG performance status was 4. Multiple BM were found in the parenchyma on both sides through MRI (**Figures 2CC, DD**). Moreover, CT of the thorax revealed enlargement of nodules in both lungs (**Figures 2C, D**). The volume of the largest tumor increased to 10.6×6.9 cm. In addition, a skin metastatic lesion on her right leg measuring 5×6 cm was detected.

Based on the high ECOG score and high predictive biomarker level, palliative monotherapy with cadonilimab10mg/kg every 21 days was administrated. After two cycles, the patient could walk. The ECOG performance status was 1. A partial response (PR) of the lung (**Figures 2E, F**), brain (**Figures 2EE, FF**) and skin metastases (Response Evaluation Criteria in Solid Tumors V1.1) had been achieved according to the CT and MRI scans. The area of the largest lung tumor decreased to 8×5 cm. The area of the largest tumor in the brain decreased to 1.5×0.8 cm. The skin metastasis also decreased in size. After five cycles of cadonilimab, a repeat chest CT scan on 20/11/2023 (**Figures 2G, H**) demonstrated a decrease lesion size. There was also a moderate decrease in the brain lesion size on MRI (**Figures 2GG, HH**). The skin metastasis disappeared. Her ECOG performance status was 0. There was a VGPR in terms of the brain and lung metastases (**Figures 2I–L, II–LL**), and a complete response (CR) in terms of the skin metastasis. The patient is still receiving maintenance monotherapy from cadonilimab every three weeks. Until the previous cycle of treatment in August 2024, the patient has a progression-free survival (PFS) of 14months after adopting cadonilimab, and has been alive for 18 months since BM was first detected.

## Discussion

BM of CC is extremely rare. Their frequency has been estimated between 0.5% and 1.2% (7, 8). The mainstays of treatments for BM are surgical resection and radiotherapy (RT), including whole-brain radiation therapy (WBRT) and SRS (9). SRS has advantages for the control of local brain metastasis and may also be used after surgery (10). Chemotherapy drugs are not typically used to treat BM because 95% of them do not pass through the intact blood-brain barrier (BBB) (11). Nevertheless, despite these therapies, the prognosis of patients of BM originating from CC remains consistently very poor. The median survival time from the diagnosis of BM to death is 2.3 months (12).

Within the past couple of years, ICIs alone and in combination with traditional treatments, have emerged as a promising treatment to combat the spread of BM and reduce the tumor burden. In the first study focusing on patients with BM from non-small cell lung cancer (NSCLC) and assessing the effects of immunotherapy, Goldberg SB (13) reported that 29.7% of PD-L1-positive patients responded to treatment of BM. This indicated that patients with BM could benefit from ICIs. The two-year survival rate of this cohort of patients (34%) exceeded that of previous reports. In the CheckMate 920 study (14) of advanced renal cell carcinoma with BM, the combination of nivolumab plus ipilimumab had encouraging antitumor efforts, with overall response rate (ORR) of 32% and a PFS of 9 months. The effectiveness of ICIs on BM derived from various cancers has demonstrated the potential of ICIs in controlling BM, and prolonging patient survival.

The mechanism by which immunotherapy exerts its effects on BM is largely unknown. Studies are in progress to develop treatments that promote antitumor immunity and reveal the mechanism underlying the antitumor effects induced by immunotherapy. The BBB can limit the access of systemic drugs delivery, which also restricts antigen presentation and immune cell infiltration. However, infiltration of brain tumors by CD8+ and CD4+ T cells has been observed (15). The underlying mechanism might include the following: 1)vascular structures lose integrity in the presence of BM, which restricts the entry of peripheral immune cell, 2) the BBB otherwise forms a “blood–tumor barrier” (BTB), which allows lymphocytes to traverse the intact BBB via chemokine axes and multistep adhesion processes (15).On the other hand, antigens specific to the central nervous system or shared between the central and peripheral nervous system are shed from BM tumor cells following tumor growth or focal treatment such as radiotherapy. these antigens might be transported to lymphoid tissue through cerebrospinal fluid and antigen presenting cells, which eventually activate a specific T-cell response (16). These findings suggest that ICIs may exert their antitumor effects on BM through T lymphocytes.

In our study, after treatment with cadonilimab, our patient achieved a rapid response of brain, lung and skin lesion, as well as a survival benefit. As a PD-1/CTLA-4 bispecific antibody, cadonilimab could activate T-cell by inhibiting CTLA-4 and reverse suppression of T-cell by targeting PD-1. These would markedly reactivate the antitumor immunity. Meanwhile, tumor infiltrating lymphocytes co-express PD-1 and CTLA-4 at much higher levels compared to normal tissues and peripheral blood cells, thus anti PD-1/CTLA4 bi-specific antibody with a preferential tumor tissue enrichment over normal tissue would contribute to enhanced efficacy and safety. Studies had observed that, cadonilimab has higher avidity of binding than PD-1 or CTLA-4 Antibody on surface with a high density of PD-1 and CTLA-4 (17)and more excellent uptake at the tumor site comparing to PD-1 or CTLA-4 targeting antibody (18). These could partially explain the efficient anti-tumor activity in this case. Moreover, the results of molecular tests revealed positive PD-L1 expression and high TMB in brain lesions which are well-established predictive biomarkers of ICI efficacy. Furthermore, RNA sequencing analysis of the TIME of brain lesions revealed favorable immune infiltration (**Figure 3**). Specifically, Alexander et al. (5) grouped the TIME into four subtypes: the immunoenriched/fibrotic subtype (IE/F) subtype, immunoenriched/nonfibrotic subtype (IE), fibrotic (F) subtype and immune desert (D) subtype. In patients, we found that both the IE/F and IE (group of our patient) subtypes exhibit high tumor immune cell invasion, that is, these are “hot” tumors that respond relatively well to immunotherapy. In addition, the GEP score based on the IFN-γ gene set associated with antigen presentation, chemokine expression, cytotoxic activity, and adaptive immune resistance pathways was applied to assess ICI response. A higher GEP was associated with superior response to ICIs (19). The activated TIME in brain lesions contributed to the good efficacy of immunotherapy. Mechanistically, radiotherapy given four months before cadonilimab likely reshapes the TIME. For instance, a TIME study of prostate cancer revealed an improved tumor-infiltrating signature after radiotherapy, which converted “cold” prostate tumors into more immunologically activated “hot” tissues (20). This finding further revealed the synergetic effect of radiotherapy and immunotherapy.

In this study, we report a rare case of CC with multiple metastases. Skin metastasis is also a preterminal sign associated with local recurrence and other metastases to distant organs (21). The clinical appearance of BM, skin metastasis and a high ECOG score indicated a poor prognosis. Our patient received palliative cadonilimab monotherapy and achieved an encouraging response and survival benefit. This could probably attribute to the dual inhibition of PD-1 and CTLA-4 of cadonilimab, and a favored TIME of targeted lesions. Although it is a case, the efficacy and inferred mechanisms of response warrant further verification, it does gave us a lot of encouragement since there is a lack of evidence on the effect of ICI in patients with CC and BM. Currently we’re summarizing clinical features, risk factors of prognosis among patients with CC. Studies on promising therapeutic regimens for CC with BM and mechanisms of response or resistance are what we want to do, and of course warrant widely investigations. We hope our case and the following studies could provide a reference for the prognosis evaluation and treatment of patients with similar presentations.

## Data Availability

The original contributions presented in the study are included in the article/supplementary material, further inquiries can be directed to the corresponding author/s.
